# The Past, Present, and Future of Comprehensive Cancer Control From the State and Tribal Perspective

**Published:** 2009-09-15

**Authors:** Sara E. Miller, Polly Hager, Kerri Lopez, Juanita Salinas, Walter L. Shepherd

**Affiliations:** Colorado Foundation for Public Health and the Environment; Michigan Department of Community Health, Lansing, Michigan; Northwest Portland Area Indian Health Board, Portland, Oregon; Texas Department of State Health Services, Austin, Texas; North Carolina Department of Health and Human Services, Raleigh, North Carolina

In 1998, the Centers for Disease Control and Prevention (CDC) funded the first comprehensive cancer control (CCC) programs in Colorado, Massachusetts, Michigan, North Carolina, Texas, and the Northwest Portland Area Indian Health Board (NPAIHB). Cancer programs were already established in these states, but with the new resources, they changed, improved, and encountered challenges. As program directors, we have been asked to reflect on these changes. The CCC community is now nationwide and has begun a new decade of collaboration to help reduce the burden of cancer. We are asking ourselves what we would like to accomplish. To answer this question, we examine past and current activities.

Before the establishment of the National Comprehensive Cancer Control Program (NCCCP), support for a coordinated approach to reduce cancer incidence and mortality through prevention, early detection, and treatment had already taken root in the 5 states and 1 tribal organization. Program leaders were already starting to understand that they needed to collaborate and coordinate their efforts. They had begun discussions about a new approach to cancer control and taken steps toward a new model. Activities were under way in these programs before CDC funding was available. Examples of these early activities include the following:

- By 1993, Colorado, with the establishment of a new breast and cervical cancer screening program, had created a CCC plan that recognized the need for collaborative efforts in cancer screening. The Colorado Cancer Coalition (1) was born, but the resources to move it forward in any substantial way were lacking.
- The Colorectal Cancer Working Group was established in Massachusetts in 1997; its focus was prevention and education (2).
- Michigan had formed the Cancer Consortium (3) in 1987 to advise the state health agency on its cancer control activities. During the 10 years that followed, the state developed policies for mammography quality assurance and passed related legislation, established a breast and cervical cancer screening program, and held a prostate cancer consensus conference.
- Starting in the late 1940s, North Carolina established a series of study committees and other temporary, cancer-specific groups. In 1993, the state passed legislation that created a permanent commission to conduct ongoing needs assessment, develop a plan to address those needs, and coordinate cancer prevention and control efforts (4).
- In 1984, the Texas legislature prepared short-term and long-term goals to reduce the burden of cancer in the state (5). The Texas Cancer Council was created to implement and coordinate these goals, which were organized into the first Texas Cancer Plan.
- The NPAIHB conducted a smokeless tobacco survey in 1987 and implemented the Northwest Tribal Tobacco Policy Project in 1989, funded by the National Cancer Institute (6). Thirty-two of 36 tribes adopted the tobacco policy in tribal facilities. In 1994, the Western Tribal Tobacco Project was funded as a national minority organization by CDC.

Because the 6 programs recognized the need to integrate activities and pool resources, they were selected to participate in a pilot study that was the foundation for the NCCCP. Although states and tribes had cancer control programs in place before they received federal funding, the new CDC resources allowed these programs more freedom to move forward with the idea of comprehensive cancer control. They were able to strengthen their own activities and to connect with sister programs and partners throughout the country.

In 2003, 4 additional tribal programs were funded (Alaska Native Tribal Health Consortium, Cherokee Nation, Fond du Lac Reservation, and South Puget Intertribal Planning Agency). A fifth (Aberdeen Area Tribal Chairmen's Health Board) was added in 2005, and a sixth (Tohono O'ohdam Nation) in 2007. Each of the tribal programs developed tools and resources specific to its own location and culture. All states, as well as these 7 tribes and tribal organizations, now have CCC plans.

A nationwide network of resources and sharing has helped make the idea of comprehensive cancer control pervasive. National partners, such as American Cancer Society, C-Change, and the National Cancer Institute, support the expansion of comprehensive cancer control by providing technical assistance, training, project support, and national policy development. Some coalitions are changing their administrative structure and moving outside state health department to have more freedom for advocacy and policy efforts. Colorado's program, for example, is now an independent CCC coalition, although it maintains a strong governmental arm.

The advancement of comprehensive cancer control has produced a growing list of topics not originally addressed in the first CCC plans. These include survivorship, pediatric cancers, genomics, and less common blood cancers. Ten years ago, the term *survivorship* was not as common as it is today, and cancer plans did not focus on it; now, most plans consider it an integral part of the cancer continuum. With the development of a vaccine for human papillomavirus, new strategies to prevent cervical cancer are being added in a growing number of cancer plans. We recognize that CCC programs must continue to be nimble and open to new science and must address technological advances in the cancer plans and programming for states.

Examples of program activities that respond to these evolving issues include the following:

- Colorado established a pediatric cancer task force to address the unique issues faced by children and their families.
- Massachusetts focused efforts on lessening prostate cancer health disparities among non-Hispanic black populations (2).
- Efforts are under way in Michigan to educate the public on the need to know their family history and to increase awareness of and access to survivorship resources.
- North Carolina created 19 work groups for specific cancer types and cross-cutting issues; survivors must make up at least 50% of the membership of each work group.
- The NPAIHB's Tribal Comprehensive Cancer Program established a strong tribal coalition, partnership network, and model training programs such as a Cancer 101 curriculum, clinicians' cancer update, and unique multidisciplinary training for tribal community workers.
- Texas created the Cancer Prevention and Research Institute of Texas, which will provide $300 million annually for cancer research and prevention programs (7).

As directors and leaders from the original NCCCP programs, we must ensure that CCC programs continue to progress. We need to clarify the role of public health departments in CCC activities and ensure that data and surveillance systems provide accurate and timely information that allows us to target interventions and measure progress. A better understanding of disparities in cancer prevention and control is needed, as is an increased focus on survivorship. We need to work with other chronic disease prevention and control initiatives to encourage behaviors that result in fewer cancers. Finally, we must ensure that screening and treatment programs are sustainable and fluid.

Having participated in the evolution of comprehensive cancer control, we believe that CCC efforts are still in an early stage; consequently, we have developed a wish list. We hope that CCC plans can be institutionalized in the states, tribes, and territories with adequate, sustained funding from public and private sources. We aim for the development of a national CCC plan that links all of the state plans cohesively, which would allow for the pursuit of a national cancer policy and would create functional communication between programs and coalitions throughout the country. Tribal and territorial partners need adequate funding from sources that respect their sovereignty.

We have grown from a few pioneering people and organizations in the late 1990s to hundreds. To move to the next level of implementing effective CCC activities, all partners need to invest in CCC coalition infrastructure. State CCC coalitions can no longer solely rely on infrastructure funding from a single source. We would like to see our other local, state, and national partners build into their future a financial and nonfinancial commitment to keep our efforts buoyant and give us the power to move forward against cancer.
